# Investigation and management of young-onset hypertension: British and Irish hypertension society position statement

**DOI:** 10.1038/s41371-024-00922-5

**Published:** 2024-06-28

**Authors:** Spoorthy Kulkarni, Luca Faconti, Sarah Partridge, Christian Delles, Mark Glover, Philip Lewis, Asha Gray, Emma Hodson, Iain Macintyre, Carmen Maniero, Carmel M. McEniery, Manish D. Sinha, Stephen B. Walsh, Ian B. Wilkinson

**Affiliations:** 1 Cambridge University Hospitals NHS Foundation Trust, Cambridge, CB2 0QQ UK; 2 Division of Experimental Medicine, University of Cambridge, Addenbrooke’s Hospital, Cambridge, CB2 0QQ UK; 3 King’s College London British Heart Foundation Centre, Department of Clinical Pharmacology, 4th Floor, North Wing, St. Thomas’ Hospital, Westminster Bridge, London, SE1 7EH UK; 4 Department of Primary Care and Public Health, Brighton and Sussex Medical School, University of Sussex, Brighton, BN1 9PH UK; 5 School of Cardiovascular and Metabolic Health, University of Glasgow, Glasgow, G12 8TA UK; 6 Deceased, formerly Division of Therapeutics and Molecular Medicine, School of Medicine, University of Nottingham, Nottingham, NG7 2QL UK; 7 Stockport NHS Foundation Trust, Stockport, SK2 7JE UK; 8 Department of Renal Medicine, Royal Infirmary of Edinburgh, National Health Service Lothian, Lothian, EH16 4SA UK; 9 William Harvey Research Institute, Barts & The London School of Medicine & Dentistry, Queen Mary University of London, Charterhouse Square, London, EC1M 6BQ UK; 10 Kings College London, Department of Paediatric Nephrology, Evelina London Children’s Hospital, Guys & St Thomas NHS Foundation Trust, Westminster Bridge Road, 3rd Floor Beckett House, London, SE1 7EH UK; 11 London Tubular Centre, Department of Renal Medicine, Royal Free NHS Trust, University College London, London, NW3 2QG UK

**Keywords:** Diagnosis, Cardiovascular diseases, Disease prevention

## Abstract

National and international hypertension guidelines recommend that adults with young-onset hypertension (aged <40 years at diagnosis) are reviewed by a hypertension specialist to exclude secondary causes of hypertension and optimise therapeutic regimens. A recent survey among UK secondary care hypertension specialist physicians highlighted variations in the investigation of such patients. In this position statement, the British and Irish Hypertension Society seek to provide clinicians with a practical approach to the investigation and management of adults with young-onset hypertension. We aim to ensure that individuals receive consistent and high-quality care across the UK and Ireland, to highlight gaps in the current evidence, and to identify important future research questions.

## Introduction

In this statement, we outline the British and Irish Hypertension Society (BIHS) recommendations for the investigation and management of adults with young-onset hypertension to enable high-quality and consistent care to be delivered across the UK and Ireland. Our framework is based on a critical review of the literature; expert opinion, where evidence is lacking; and a survey among secondary care hypertension specialist physicians working across the UK and Ireland (Table 1). We compare the prevalence of hypertension among young adults in the UK and internationally and review the underlying mechanisms that contribute to raised blood pressure (BP). We highlight the gaps in current evidence and identify important future research questions.

**Table 1:** Results of a BIHS membership survey^a^ on investigations routinely offered to all new patients with young-onset hypertension referred to secondary care.

Investigation	% of patients routinely offered this investigation
Clinic BP	96
ABPM	54
Home BP Monitoring	65
ECG	62
ECHO	54
Renal Ultrasound	42
MRA aorta/renal/adrenal	19
Urine albumin-creatinine ratio	85
24 h urine collection for normetadrenaline, metadrenaline, sodium, potassium and creatinine	69
**Blood tests**	
Aldosterone/renin ratio	92
Plasma metanephrines	54
Electrolytes	100
Creatinine/eGFR	100
Cholesterol profile	92
Full Blood Count	92
Additional tests that clinicians reported routinely requesting for all patients with young-onset hypertension included: 24 h urinary free cortisol, 24 h albuminuria, liver function tests, calcium, echocardiography, weight and height, Epworth and STOP-Bang scoring and exercise BP.	

*ABPM* Ambulatory blood pressure monitoring, *BIHS* British and Irish Hypertension Society, *BP* Blood pressure, *ECG* Electrocardiography, *ECHO* Echocardiography, *eGFR* Estimated glomerular filtration rate, *MRA* Magnetic Resonance Angiography.

^a^In September 2023 an online survey was emailed to BIHS members to help us understand what investigations are routinely offered to young people referred to specialist secondary care hypertension clinics. We received 26 replies from members working across England, Wales, Scotland and Ireland. The results of the survey are reported here.

In this statement we define young-onset hypertension as the onset of hypertension in individuals aged < 40 years. National, European and International hypertension guidelines recommend adults with young-onset hypertension are referred to hypertension specialists to exclude secondary causes of hypertension, assess end-organ damage (EOD) and optimise therapeutic regimens [1–6].

We believe the prompt investigation and management of young-onset hypertension is important for 2 reasons:

- Early detection and treatment of the secondary causes of hypertension can be curative.
- Early normalisation of BP may reduce damage to the vasculature and other organs, thus reducing lifetime cardiovascular risk.

## Epidemiology

Although there is a clear relationship between BP and cardiovascular and renal morbidity and mortality, independent of age, the ‘seeds’ of hypertension are often sown during childhood [7–13]. As hypertension-mediated EOD is not fully reversible [14], it is important to promptly identify and manage those with young-onset hypertension to mitigate their increased lifetime risk of cardiovascular disease (CVD).

Estimates of the prevalence and incidence of hypertension vary amongst young adults, depending on diagnostic thresholds and the population studied, making direct comparisons difficult. In England, 9% of young adults (aged 16–44 years) were reported as having hypertension in 2021, defined as BP ≥ 140/90 mmHg or taking antihypertensive medication [15]. Prevalence data from the United States for 2017–18 suggests 20.6% of young adults (aged 18–39 years) have hypertension defined as BP ≥ 130/80 mmHg or on antihypertensive medication [16]. A recent meta-analysis reported the global prevalence of hypertension (defined as BP ≥ 120/80 mmHg or ≥ 90th centile for age, sex and height) among those aged under 19 years was 4.0% [95% CI 3.3%–4.8%] and had increased over the reported period (1994 to 2018) [17].

The 2 year incidence of new onset hypertension (BP ≥ 160/95 mmHg or starting treatment) among young adults (30–39 years) in the Framingham cohort was 3.3% in men and 1.5% in women [18]. The Coronary Artery Risk Development in Young Adults (CARDIA) study reported a 10 year incidence of hypertension ( ≥ 130/85 mmHg or on antihypertensive medication) in young people (18-30 years at baseline) of 16.4% and 13.1% in black men and women respectively; and 7.8% and 3.2% in white men and women respectively [19]. This is consistent with pooled data from the Health Survey for England 2011−19 which reported the prevalence of hypertension was highest among black Caribbean adults (39%) and lowest among adults from white other (13%) and Chinese backgrounds (8%) [20].

Hypertension is also associated with other social and demographic factors, including socio-economic deprivation and obesity (body mass index [BMI] ≥30 kg/m^2^). In England, the reported prevalence of hypertension was 23% in the least deprived areas, rising to 40% in the most deprived areas [15]. Obesity is a global problem, and the worldwide prevalence has nearly tripled between 1974 and 2016 [21]. In England, obesity currently affects 10% of those aged 16 to 24 years rising to 28% among those aged 35 to 44 years [15].

Estimates of the prevalence of secondary hypertension among young adults vary considerably depending on the population studied and extent of specialist investigations undertaken [22]. However, coarctation of the aorta, phaeochromocytoma, Cushing’s syndrome and thyroid disease are rare, with a prevalence among people with young-onset hypertension of 1% or less. In contrast, up to 10% may have primary hyperaldosteronism, renal artery stenosis (more commonly fibromuscular dysplasia [FMD], Takayasu arteritis or neurofibromatosis rather than atherosclerosis), or renal parenchymal disease [22]. Importantly, early identification and treatment of secondary causes of hypertension improves patient outcomes [2–6].

Box 1 Young-onset hypertension (<40 years at diagnosis)

- **Estimated overall prevalence** (BP≥140/90 mmHg or antihypertensive treatment): ~9%
- **Key risk factors**: black, male, high salt diet, overweight, low socio-economic status
- **Estimated prevalence of secondary causes**: ~ 10%

## Pathophysiology of hypertension in young adults

Although often viewed as a single condition, hypertension encompasses several distinct patterns of BP elevation such as isolated systolic (ISH), systolic diastolic (SDH) and isolated diastolic (IDH) hypertension, each with different underlying haemodynamic mechanisms. ISH is the most common form of hypertension in young adults. It is more common in males and those with a higher BMI [23–25]. Elevated cardiac output, driven by an increased stroke volume, is the key underlying haemodynamic characteristic. A smaller number of young subjects with ISH have a normal stroke volume but increased aortic pulse wave velocity – a measure of aortic stiffness [24, 25]. Indeed, a recent meta-analysis of the haemodynamics of primary hypertension in children and adults concluded that the main haemodynamic alteration in primary hypertension (including obesity-related hypertension), in both children and young to middle-aged adults, is an elevation of cardiac output [26].

SDH and IDH are the most common hypertensive phenotypes in young females, and are characterised by increased peripheral vascular resistance and, in some cases, elevated aortic stiffness [24]. Cardiac output may also be raised, but because of high heart rate rather than an increased stroke volume [27].

Overall, these findings are consistent with the view that hypertensive young males display a predominantly ‘cardiac’ BP phenotype, whereas hypertensive young females have a predominantly ‘vascular’ BP phenotype. These differences may reflect divergent pathophysiology and may require alternate therapeutic approaches, but this remains to be tested formally in prospective research studies.

## Clinical assessment

### Medical history

A detailed medical history should be obtained including the elements outlined in Table 2. It is important to specifically ask about substances that may cause hypertension including:

- Concurrent medications
- Contraceptive medication
- Hormone substitutes
- Over-the-counter preparations
- Herbal/traditional remedies
- Recreational drugs

**Table 2:** Medical history.

**Presentation of Hypertension** • Time of first diagnosis of hypertension • Current/past antihypertensive medications including intolerances • Adherence to therapy • Family history of hypertension, CVD, stroke, kidney disease • History of erectile dysfunction • Previous hypertension in pregnancy/preeclampsia/foetal growth restriction
**Concurrent conditions** • Concurrent conditions, noting risk factors for CVD e.g., hypercholesterolaemia, PCOS • Sleep history, snoring, sleep apnoea (where applicable consider partner’s view)
**Concurrent medications (noting drugs that may increase BP)** •Prescribed medication noting combined oral contraceptive pill or implants, hormone substitutes, steroids, nonsteroidal anti-inflammatory drugs, vascular endothelial growth factor inhibitors, tyrosine kinase inhibitors, tricyclic antidepressants, selective serotonin noradrenaline reuptake inhibitors, dexamphetamine and methylphenidate may increase BP • Over-the-counter medications noting herbal remedies and decongestants may increase BP • Recreational drugs/substance abuse including but not limited to cocaine, amphetamines, ecstasy and anabolic steroids
**Lifestyle** • Tobacco smoking history: current/ex/never (pack years) • Dietary history: fruit and vegetable intake, bread and breakfast cereal intake, salt intake, alcohol consumption, liquorice • Physical activity per week • Weight gain or loss

*BP* Blood pressure, *CVD* Cardiovascular disease, *PCOS* Polycystic ovary syndrome.

Patients should be asked about symptoms suggestive of secondary causes of hypertension, noting that absence of symptoms does not adequately exclude secondary causes (Table 3). It is important to take an obstetric history, focusing on occurrence of hypertension in pregnancy and foetal growth restriction, which are risk factors for the development of hypertension post-partum and are associated with increased CVD risk [28]. Patients should also be asked about symptoms of obstructive sleep apnoea and clinicians may wish to consider the use of an assessment tool, such as the STOP-Bang questionnaire, to assess risk [29].

**Table 3:** Signs and symptoms suggestive of secondary causes of hypertension.

Pheochromocytoma	Repetitive episodes of palpitation, (usually slow) headache, pallor and anxiety, angor animi
Hyperaldosteronism	History of spontaneous or diuretic-provoked hypokalaemia, episodes of muscle weakness and tetany
Hypothyroidism	History of increased diastolic BP, fatigue, weight gain, dry skin, hair loss, constipation and muscle weakness
Hyperthyroidism	History of increased systolic BP, tremor, anxiety, sweating, weight loss, diarrhoea and heat intolerance
Cortisol/glucocorticoid excess (Cushing’s)	Truncal obesity with thin limbs, buffalo hump, striae, round (moon) face, thin frail skin that bruises easily, acne
Neurofibromatosis	Café-au-lait skin patches, fibromas, axillary freckles
Acromegaly	Facial changes, enlargement of hands and feet, sweating
Chronic kidney disease	Weight loss, haematuria, itching, ankle swelling, shortness of breath
Pre-eclampsia	History of BP elevation in previous or current pregnancy
Renal scarring	History of repetitive renal/urinary tract disease/urinary tract infection/stones

*BP* Blood pressure.

### Family history

A detailed family history should be obtained including occurrence of FMD or other renal diseases, phaeochromocytoma, neurofibromatosis, thyroid disease, hyperaldosteronism, and monogenic hypertension. A family history of early-onset hypertension, sudden death, stroke or myocardial infarction (MI) at a young age ( < 60 years) in a first-degree relative may suggest the presence of secondary causes and/or monogenic hypertension. Patients should also be encouraged to ask relatives and update their records at subsequent visits.

### Physical examination

**Fig. 1:**
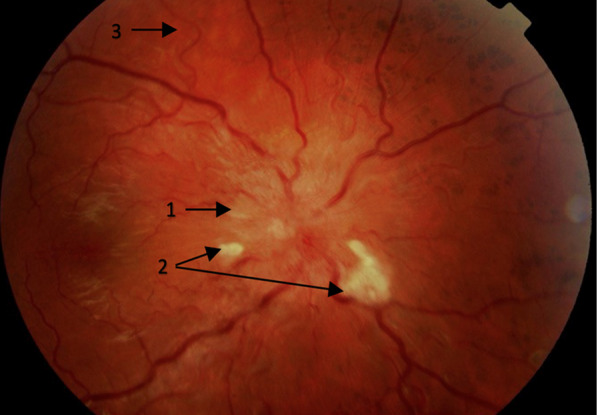
Retinal fundus showing hypertension-mediated end organ damage. 1 = Blurred disc margins and papilloedema, 2 = Cotton wool spots, 3 = Generalized arteriolar attenuation and tortuosity.

A detailed physical examination should be undertaken, focusing on signs of secondary hypertension and evidence of EOD (Tables 3 and 4), including:

- Observe for signs of Cushing’s syndrome, acromegaly and assess thyroid status.
- Palpate radial and femoral pulses to detect radio-radial or radio-femoral delay of aortic coarctation.
- Auscultation of heart for murmurs and carotid, renal and femoral arteries for bruits.
- Palpate the kidney for signs of enlargement evident in polycystic kidney disease.
- Conduct fundoscopy to assess EOD, with mydriasis if needed. Examination of the retinal fundus is particularly useful in young-onset hypertension as it may point towards chronic subclinical EOD (Fig. 1), particularly where evidence for a sustained rise in BP is inconsistent (e.g. white coat hypertension). Papilloedema may be present in patients with benign intracranial hypertension.

**Table 4:** Assessment of End Organ Damage (EOD).

Organ	Clinical diagnosis associated with EOD	Subclinical findings suggestive of EOD
Heart	Heart failure (HFpEF, HFrEF), Coronary artery disease Arrhythmias (especially Atrial fibrillation)	Left ventricular hypertrophy Left ventricular systolic or diastolic dysfunction Dilated atria Raised BNP or NT pro-BNP
Kidney	Chronic kidney disease	Microalbuminuria/Proteinuria Declining eGFR Reduced cortical thickness or other features of renal disease
Eye	Visual impairment	Hypertensive retinopathy (Fig. 1) (haemorrhages, microaneurysms, hard exudates, cotton wool spots)
Brain	Cerebrovascular accident/ Transient ischaemic attack	Lacunes, microbleeds or large white matter hyperintensities on MRI Carotid artery atheroma
Blood vessels	Peripheral vascular disease, Aortic aneurysm	Mild thoracic aortic dilatation Aortic stiffening Reduced ankle-brachial index Atherosclerotic plaques

*BP* Blood pressure, *BNP* Brain natriuretic peptide, *eGFR* Estimated glomerular filtration rate, *HFpEF* Heart failure with preserved ejection fraction, *HFrEF* Heart failure with reduced ejection fraction, *MRI* magnetic resonance imaging, *NTpro-BNP* N-terminal pro BNP.

### Blood pressure measurement

Office/clinic BP measurement is easily accessible as an initial screening tool. The mandatory minimum is to use a BIHS-validated device (https://bihsoc.org/bp-monitors/) with an appropriately sized cuff (the bladder should encompass 80-100% arm circumference) applied to the upper arm with the subject seated, silent, resting with arm supported at heart height and back supported, bladder empty [3]. Most BP screening is performed using semi-automated oscillometric monitors. There are currently no data on the effect of age on the accuracy of BP measurement using such monitors, although some have been specifically validated for paediatric subjects and pregnant women [30, 31].

A diagnosis of hypertension and subsequent management decisions should not be made on a single occasion due to marked individual BP variability [32], except in the context of a hypertensive crisis [33]. Office/clinic BP should be repeated at least three times (averaging the final 2 readings) at one-to-two-minute intervals on several different occasions [3]. If the BP is raised then a diagnosis of hypertension should be confirmed by ambulatory, or home BP monitoring.

At the first visit, BP should be measured in both arms and the arm with the higher readings used for subsequent BP measurement. In individuals with postural symptoms or risk factors for autonomic neuropathy (e.g. diabetes), BP should also be recorded supine and standing (after at least 1 min). A diagnosis of postural hypotension can be considered if systolic BP falls by ≥20 mmHg and/or diastolic BP falls by ≥10 mmHg [3, 34].

### Unattended BP measurement

Unattended clinic/office BP measurements (recorded when the patient is seated alone and unobserved) are ~5–15 mmHg lower than those obtained by conventional office BP measurement and are thought to correspond better to ambulatory BP measurements than observed (i.e. usual) clinic BP [35–38]. As there is currently limited evidence on the prognostic value of unattended office BP measurements [39], we advise therapeutic decisions are based on observed clinic/office BP and/or ambulatory/home BP targets.

### Ambulatory/home BP measurement

Ambulatory/home BP monitoring improves the accuracy of BP assessment and is mandatory to confirm a diagnosis of hypertension. Ideally, this will have been performed before referral to a secondary care hypertension specialist [1]. However, ambulatory/home monitoring may need to be repeated if there is a significant difference between clinic/office and home/ambulatory readings, particularly in the absence of target organ damage, and in cases of apparent resistant hypertension.

Box 2 BP thresholds for further investigation and treatment

**Table:** 

• Attended office/clinic BP	≥ 140/90 mmHg
• Ambulatory day/awake mean BP	≥ 135/85 mmHg
• Ambulatory night/asleep mean BP	≥ 120/70 mmHg
• Ambulatory 24hr mean BP	≥ 130/80 mmHg
• Home mean BP	≥ 135/85 mmHg

### White coat and masked hypertension

**Fig. 2:**
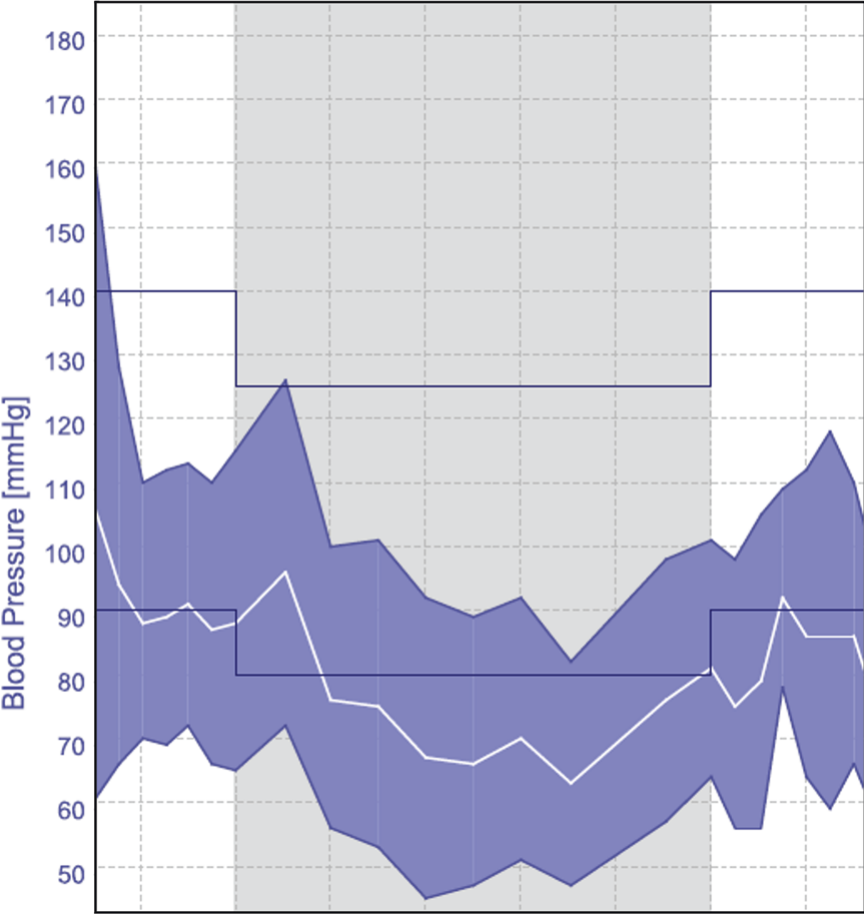
Ambulatory blood pressure monitoring demonstrating white coat hypertension. Ambulatory Blood Pressure Monitoring (ABPM) from a patient with young-onset hypertension demonstrating white coat effect with office/clinic BP and the first reading on ABPM > 140/90 mmHg but normal day, night (grey shaded area) and total BP averages.

White coat hypertension i.e. high office/clinic BP with normal ambulatory/home BP (Fig. 2), is more common among women and is a predictor of developing sustained hypertension and CVD. Individuals with white-coat hypertension should be closely monitored [40–43].

**Fig. 3:**
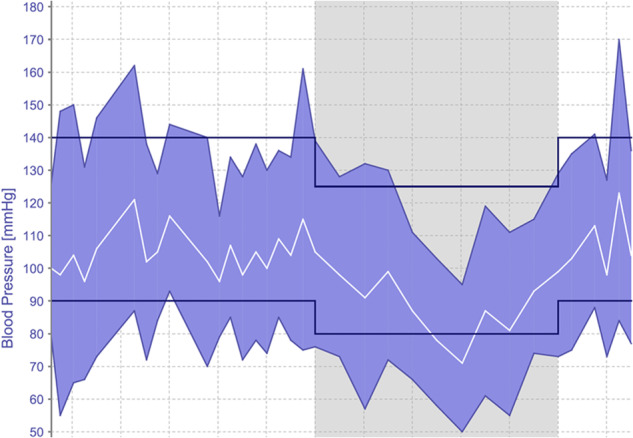
Ambulatory blood pressure monitoring demonstrating masked hypertension. Ambulatory Blood Pressure Monitoring (ABPM) from a patient with young-onset hypertension demonstrating masked hypertension with office/clinic BP and the first reading on ABPM < 140/90 mmHg but elevated daytime average BP. The grey shaded area denotes night-time readings.

Masked hypertension i.e. normal office/clinic BP with elevated ambulatory/home BP (Fig. 3), is more common in men, smokers and those with higher BMI and SBP [44, 45]. If masked hypertension is demonstrated on ambulatory/home monitoring, therapeutic decisions should be based on out-of-office BP targets.

Higher rates of both white coat hypertension (15–25%) and masked hypertension (10–20%) have been reported among those with secondary hypertension [32, 46]. However, it is important to note that many of the studies relating to the prognostic value of white coat and masked hypertension are very small in comparison to data on office/clinic BP and outcomes, and further data are required.

### Urinalysis

Albuminuria and/or haematuria may indicate renal parenchymal disease or hypertension-associated EOD in the absence of urinary tract infection and should be investigated further (Tables 4 and 5). Formal urinary albumin: creatinine ratio (UACR) is recommended as a more robust measure of albuminuria than dipsticks. Pregnancy should be excluded in women with child-bearing potential.

**Table 5:** Consider the following investigations for all adults with young-onset hypertension.

Investigation of EOD and assessment of cardiovascular risk (all patients)	
Clinic/Office BP	
ABPM/HBPM	
ECG	
ECHO (to assess EOD and coarctation of aorta)	
Blood tests to include: Creatinine, eGFR, TC, TG, HDL, TC:HDL, LDL, NHDLC, Glucose, HBA1c, Liver function (combine with those listed below)	
Fundoscopy	
Height, weight and waist circumference	
Office dipstick urinalysis	
**Investigations for secondary hypertension**	
**Blood tests (all patients)** Sodium, Potassium, Urea, Bicarbonate, Corrected Calcium, Phosphate TSH Full blood count Renin, Aldosterone (Aldosterone/renin ratio) Plasma metanephrines (or urine)	**Additional blood tests (specific clinical scenarios):** Chloride, Magnesium ESR, CRP PTH, Vitamin D (patients with calcium abnormalities) Lp(a) (if strong family history of premature CVD) Renal vasculitis screen (patients suspected to have renal disease based on urine protein and/or blood, inflammatory markers, creatinine and/or renal imaging) Dexamethasone suppression test (patients with clinical suspicion of Cushing’s and/or adrenal adenoma on imaging)
**Urine tests (all patients)** Urine sample for ACR and/or PCR	**Additional urine tests (specific clinical scenarios):** Urine for drug adherence screen (resistant hypertension, apparent lack of response, variability in BP response) Urine for drugs of abuse (concomitant tachycardia, symptoms suggestive of catecholamine surge)
**24-hour urine test (all patients)** Creatinine and electrolytes (Sodium, Potassium) Normetadrenaline, Metadrenaline (if plasma test is not available)	**Additional 24-hour urine test (specific clinical scenarios):** Aldosterone and Cortisol (in patients suspected primary aldosteronism and/or Cushing’s disease) Urine for Calcium, Phosphate, Chloride, Magnesium (in patients with a history of recurrent renal stones, renal genetic tubular disorder) 24-hour urine 5HIAA (suspected carcinoid syndrome)
**Imaging (all patients)** Renal imaging (e.g. ultrasound) *Note: FMD can only be excluded by formal angiography*	**Additional imaging (specific clinical scenarios)** MRA/CTA Aorta/Renal (may also allow for simultaneous adrenal imaging and detection of FMD, adrenal adenoma/ indeterminate adrenal lesions, coarctation of aorta)
**Genetic Testing Referrals** are triaged by the Genomic Laboratory to prioritise individuals where a genetic or genomic diagnosis will guide management for that individual or family. Please refer to the National Genomics Test directory: https://www.england.nhs.uk/publication/national-genomic-test-directories/ and https://www.england.nhs.uk/genomics/genomic-laboratory-hubs/.	
**Additional Investigations** should be performed as indicated to confirm clinical/ subclinical EOD and inform management decisions.	

*ABPM* Ambulatory blood pressure monitoring, *ACR* Albumin:Creatinine ratio, *BP* Blood pressure, *CRP* c-reactive protein, *CTA* Computed tomography angiography, *ECG* Electrocardiography, *ECHO* Echocardiography, *eGFR* Estimated glomerular filtration rate, *ESR* Erythrocyte sedimentation rate, *FMD* Fibromuscular dysplasia, *HBA1c* Haemoglobin A1c, *HDL* High density lipoprotein, *HBPM* Home blood pressure monitoring, *LDL* Low density lipoprotein, *NHDLC* Non high density lipoprotein cholesterol, *MRA* Magnetic Resonance Angiography, *PCR* Protein Creatinine ratio, *PTH* Parathyroid Hormone, *TSH* Thyroid Stimulating Hormone, *TC* Total Cholesterol, *TG* Triglyceride, *5-HIAA* 5-Hydroxyindoleacetic acid.

### Height, weight and waist measurement

Height and weight should be measured, and BMI calculated. Waist size should be measured to inform total cardiovascular risk.

### Further investigations

Consider the investigations outlined in Table 5 for all adults with young-onset hypertension to assess EOD, cardiovascular comorbidities and secondary causes of hypertension. Whilst conducting all investigations may seem overly comprehensive, missing a potentially curable form of secondary hypertension may have a substantial deleterious impact given that patients face a lifetime of antihypertensive treatment and increased cardiovascular risk.

Biochemical investigations including renin, aldosterone and plasma or 24 h urine metadrenalines (metanephrines) aid diagnoses of primary aldosteronism and phaeochromocytoma, respectively. Both plasma and urine fractionated metadrenalines are acceptable for routine screening, with some suggestion that plasma may have a higher specificity [47].

Although 24-hour urine collection may be considered cumbersome by some patients, complete collection allows estimation of daily salt intake and interpretation of plasma renin/aldosterone [48]. Cortisol and aldosterone can also be done on the same urine sample and can help exclude Cushing’s syndrome and hyperaldosteronism.

Although hyper-reninaemic hyperaldosteronism is a classical finding in cases of renal artery stenosis of various aetiologies including FMD, renin and aldosterone may be normal in some patients. Screening is usually via computed tomography (CT) angiography or magnetic resonance (MR) angiography. Digital subtraction angiography provides a definitive diagnosis and the option of therapeutic angioplasty [49, 50].

Coarctation of the aorta is a diagnosis usually made in childhood (60% of the cases) and accounts for 5-8% of congenital heart disease [51]. When asymptomatic or non-severe, coarctation of the aorta may remain undiagnosed and persist into adulthood. Patients may present with resistant hypertension as adults, but often the diagnosis is delayed and missed. A chest radiograph may appear normal, and classical clinical signs of radio-femoral delay or murmur may be absent. In such cases, an echocardiography is the screening investigation of choice although images may still be suboptimal, especially in a post-ductal coarctation of the aorta [52]. Aortic imaging modalities such as CT and MR imaging (which will also help to assess the anatomy of the collaterals) will confirm the diagnosis.

## Monogenic hypertension

Monogenic hypertension arises from specific gene mutations [53]. These syndromes are likely to present early in life so, although rare, will be relatively over-represented in those with young-onset hypertension. They pose a diagnostic challenge due to the complexity of their clinical and biochemical phenotypes, and significant heterogeneity in severity and presentation. However, the following factors should increase clinical suspicion:

- Young-onset hypertension
- Low renin hypertension
- Persistent electrolyte abnormalities (specifically potassium and bicarbonate)
- Brachydactyly and hypertension (associated with PDE3A mutation)
- Disorders of adrenal steroids (e.g. congenital adrenal hyperplasia)
- Family history of monogenic hypertension, young-onset hypertension, sudden death, stroke or MI at a young age ( < 60 years) in a first degree relative

Accurate diagnosis is important as these conditions are highly sensitive to specific drug regimens due to their specific molecular aetiology (Table 6). In England, genetic testing referrals are triaged by the Genomic Laboratory to prioritise individuals where a genetic or genomic diagnosis will guide management for that individual or family. Please refer to the National Genomics Test directory: https://www.england.nhs.uk/publication/national-genomic-test-directories/ and https://www.england.nhs.uk/genomics/genomic-laboratory-hubs/.

**Table 6:** Examples and recommended therapy for monogenic hypertension.

Condition	Recommended Treatment
Liddle syndrome	Amiloride
Apparent mineralocorticoid excess	Spironolactone
Gordon syndrome	Thiazide
Geller syndrome	Amiloride (spironolactone is contraindicated)
Glucocorticoid-remediable aldosteronism (familial hyperaldosteronism type 1)	Glucocorticoids (or steroidal MRA)
Familial hyperaldosteronism type 2,3,4	Steroidal MRA (Consider adrenalectomy in patients who do not respond to steroidal MRA)
PASNA syndrome (primary aldosteronism, seizures and neurological abnormalities)	Steroidal MRA and CCB
11beta-hydroxylase deficiency	Glucocorticoids
17alpha-hydroxylase deficiency	Glucocorticoids

*CCB* Calcium channel blocker, *MRA* Mineralocorticoid receptor antagonist.

## Treatment

There are two well-established strategies to lower BP, lifestyle interventions and pharmacological treatments. Outcome-based randomised controlled trials (RCTs) have clearly shown the benefit of pharmacological BP reduction for the prevention of cardiovascular events and reduction in all-cause mortality [54]. However, such studies have largely included middle-aged and older adults, and no such data are available in young adults. As a result, recommendations for life-long pharmacological treatment for younger and lower-risk patients are based on considerable extrapolation.

Despite these uncertainties, there is a consensus that lifestyle interventions should be recommended for all individuals with hypertension [1–6]. This is supported by evidence that the cardiovascular system may be able to remodel during childhood when hypertension is less likely to have been long-standing [55–57].

There is controversy about whether younger adults with uncomplicated stage 1 hypertension should receive pharmacological treatment. In contrast to clear evidence among older subjects [58, 59], the National Institute for Health and Care Excellence (NICE) currently recommends an approach based on the estimation of CVD risk in younger subjects *without* established CVD, renal disease or diabetes [3]. There are 3 limitations of this approach:

- Prediction models for the 10-year risk of CVD underestimate the risk in young subjects and don’t provide information on life-time risk.
- Diastolic BP may have a greater risk-predictive value than systolic BP alone in younger adults [60].
- The value of hypertension-mediated EOD in refining risk estimation has, to date, not been well defined (Table 4).

**Fig. 4:**
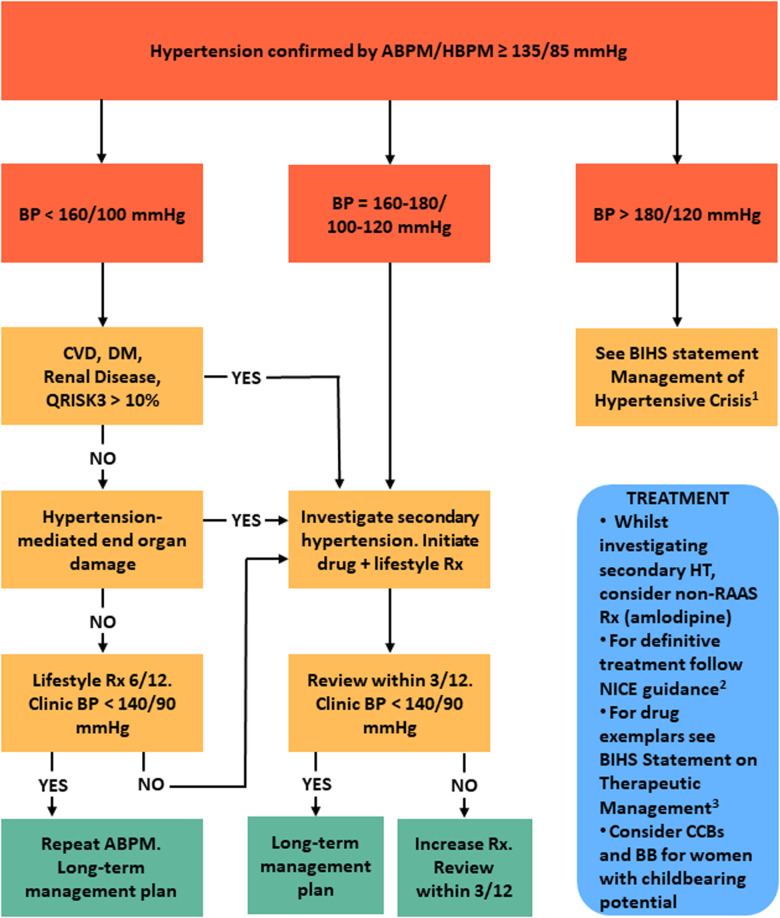
Investigation and treatment of young-onset hypertension. ABPM Ambulatory blood pressure monitoring, BB Beta blocker BP Blood pressure, CVD Cardiovascular disease, CCB Calcium channel blocker, DM Diabetes mellitus, HBPM Home blood pressure monitoring, HT Hypertension, RAAS Renin-angiotensin-aldosterone system, Rx Treatment. [1] Kulkarni S et al., J Hum Hypertens. 2023;37(10):863–79 [2] NICE guideline NG136: https://www.nice.org.uk/guidance/ng1362022 [3] Lewis P. et al., J Hum Hypertens. 2024;38(1):3–7.

As a result, young adults with hypertension often do not receive “early” pharmacological treatment and must wait until their cardiovascular risk threshold crosses 10%. However, early treatment may prevent the development of more severe hypertension and EOD. This is particularly important as later treatment may not fully reverse EOD, particularly in the vasculature and kidney [14, 61]. To mitigate this, we recommend a more pragmatic approach presented in Fig. 4, which more closely aligns with guidelines from other international hypertension societies [2, 4–6].

In cases of markedly elevated BP ( > 180/120 mmHg), an urgent assessment is needed and recommendations from the BIHS guidance on the management of hypertension crisis should be followed [33].

Among subjects with BP between 160 and 180/100–120 mmHg pharmacological interventions alongside lifestyle measures should be started promptly. If secondary causes of hypertension have not been excluded, non renin-angiotensin-aldosterone system (RAAS) interfering drugs (such as amlodipine) are the preferred choice as a temporary treatment, whilst long-term intervention should follow NICE recommendations [1, 3]. Among women of childbearing potential it is important to discuss plans for future pregnancy and contraception use and, where appropriate, the potential detrimental effects of angiotensin converting enzyme inhibitors/angiotensin II receptor antagonists in pregnancy. An informed decision can then be made about whether drugs that can be routinely used during pregnancy (such as modified release nifedipine, amlodipine or beta blockers) should be used in place of RAAS-blocking drugs [62].

Pharmacological treatment for hypertension is also recommended in individuals with BP values < 160/100 mmHg at moderate-high risk (established CVD, diabetes, renal disease, QRISK3 > 10%).

In subjects at low risk and with BP < 160/100 mmHg, the decision-making process should be guided by the assessment of the EOD (Table 4). In patients with evidence of EOD, pharmacological treatment should be commenced promptly. In the absence of EOD, we recommend optimisation of lifestyle measures, focusing on weight optimisation and reduction in dietary salt and alcohol for an interim period of 3 to 6 months. If there is normalisation of *clinic* BP following this, a 24 h ambulatory or home BP monitoring should be considered to confirm normotension and exclude masked hypertension. If BP remains elevated after 6 months, then we recommend starting pharmacological therapy following a discussion of the risk benefits with the patient.

Once treatment is started, BP should be reassessed within 3 months with a BP target below < 140/90 mmHg (ambulatory < 135/85 mmHg) [3]. In more complex clinical situations (such as patients with diabetes, aortic dissection, or post-stroke) BP targets recommended by NICE should be followed [3]. The use of home monitoring may allow targets to be reached more quickly if a plan is made to up-titrate or commence additional drugs based on HBPM readings.

In discussion with the patient, lower BP targets may be considered given the lifetime risk faced by younger patients. Although this recommendation is not directly supported by RCTs, extrapolation of outcome data from higher-risk cohorts supports a more stringent BP control for long-term CV prevention in populations at relatively low absolute CV risk [63–67]. Moreover, the average population BP in young individuals is considerably lower than in middle-aged and older age groups [15]. Thus ‘normalisation’ of BP requires more aggressive targets. However, the benefits of this approach remain to be tested in randomised controlled trials. If lower targets are desirable, then single-pill combinations may be helpful in achieving this with the benefits of a reduced pill burden and better adherence [68].

The NICE adult hypertension guidelines were informed by an economic model that suggested starting patients with stage 1 hypertension on pharmacotherapy was cost-effective for men regardless of age and 10-year cardiovascular risk and for women > 60 years [3, 69]. However, the model did not include patients under 40 years of age and did not consider other healthcare costs such as those associated with hypertension in pregnancy. The cost of investigations and management for younger people includes upfront expenses for blood tests, imaging and specialist consultations, which must be balanced by the substantial costs of uncontrolled hypertension leading to EOD. The costs to patients themselves including prescriptions, health/travel insurance and psychological factors should also be considered depending on the health care setting. Early intervention can potentially lead to better health outcomes and overall reduction in the financial burden of managing the consequences of uncontrolled hypertension [70–72].

Box 3 Lifestyle interventions to reduce blood pressure and cardiovascular risk [1–6]

1. **Reduce salt to <6g day**. Pre-prepared foods like bread, breakfast cereals and ready meals may contain high salt. Check labels when shopping and avoid adding extra salt to food.
2. **Maintain a healthy weight**. For people that are overweight, weight loss is associated with a reduction in blood pressure.
3. **Incorporate exercise**. 30 minutes of moderate aerobic exercise 5 times a week and resistance training 2 to 3 times a week.
4. **Eat a balanced diet**. Include at least 5 portions (400g) of fruit and vegetables daily.
5. **Alcohol in moderation**. Current guidelines recommend up to 14 units a week for men and women. Avoid binge drinking and encourage alcohol-free days.
6. **Caffeine in moderation**. Be mindful that caffeine is in cola and energy drinks as well as tea and coffee.
7. **Stop smoking**. To reduce cardiovascular risk.

## Transition from paediatric to adult services

We note that in those with childhood-onset hypertension, adolescence and transition to adult services are particularly challenging periods for young people, their families and clinical teams [13]. Currently, there are no nationally accepted models regarding the transition of health care for hypertensive children, but good practice models exist and should be considered to ensure continuity of care [13, 73].

Box 4 Research Recommendations

1. Is BP ≥140/90 mmHg an appropriate threshold to initiate pharmacological treatment in younger adults, or should it be lower?
2. Is the current NICE treatment target (clinic BP <140/90 mmHg) too high and should the treatment target in younger people reflect age-specific population BP averages e.g. <120/80 mmHg?
3. Do we need sex-based thresholds and targets, as women have lower population BP?
4. Should we support weight loss, where appropriate, with pharmacotherapy (e.g. GLP1 agonists) before starting antihypertensive treatment?
5. What is the most effective drug for young people, especially those in the early, high cardiac output phase of hypertension, and should BP treatment be sex specific given the different underlying haemodynamic mechanisms?
6. What is the cost-effectiveness of comprehensive screening for the secondary causes of hypertension among those with young-onset hypertension in the UK?
7. What can developments in imaging technology (e.g. lower cost, more efficient MRI) offer for comprehensive organ screening?
8. What is the cost-effectiveness of specialist hypertension clinics for young people?

## Conclusion

In summary, national and international studies suggest there are large numbers of young people with raised BP that, left untreated, are likely to suffer from avoidable premature morbidity and mortality. Current guidelines for initiating antihypertensive treatment are based on 10-year cardiovascular risk. As risk scores are strongly determined by age, and evidence from antihypertensive treatment trials are based on older individuals, younger people may not be accessing appropriate therapies to meet their needs, and current guidelines are therefore inherently ageist.

This BIHS statement provides a practical approach to the investigation and management of young-onset hypertension to enable consistent and high-quality care to be delivered across the UK and Ireland and poses important future research questions to improve patient outcomes.

## Summary

### What is known about this topic

- Management, investigations and treatment for young-onset hypertension varies across the globe.
- Traditionally thresholds for initiating antihypertensive treatment are based on 10-year cardiovascular risk estimation, which significantly disadvantages young people, who face a lifetime of increased risk.
- Not treating hypertension in young people potentially exposes them to accruing irreversible end-organ damage over time.

### What this study adds

- This BIHS statement provides a practical framework for investigating and managing young-onset hypertension.
- We emphasise the need to personalise management in young people particularly with regard to risk estimation and pharmacological treatment, while avoiding over-treatment.
- We emphasize the need for robust prospective evidence for treatment (pharmacological and nonpharmacological) and BP targets in young-onset hypertension.

## Data Availability

All data generated and analysed for the development of this statement are included in this published article.

## References

[1] Lewis P, George J, Kapil V, Poulter NR, Partridge S, Goodman J (2024). Adult hypertension referral pathway and therapeutic management: British and Irish Hypertension Society position statement. J Hum Hypertens.

[2] Mancia G, Kreutz R, Brunstrom M, Burnier M, Grassi G, Januszewicz A (2023). 2023 ESH Guidelines for the management of arterial hypertension The Task Force for the management of arterial hypertension of the European Society of Hypertension: Endorsed by the International Society of Hypertension (ISH) and the European Renal Association (ERA). J Hypertens.

[3] National Institute for Health and Care Excellence. Hypertension in adults: diagnosis and management. NICE guideline [NG136]. Published 28 August 2019, Last updated: 21 November 2023. https://www.nice.org.uk/guidance/ng1362022.

[4] Unger T, Borghi C, Charchar F, Khan NA, Poulter NR, Prabhakaran D (2020). 2020 International Society of Hypertension Global Hypertension Practice Guidelines. Hypertension.

[5] Williams B, Mancia G, Spiering W, Agabiti Rosei E, Azizi M, Burnier M (2018). 2018 Practice Guidelines for the management of arterial hypertension of the European Society of Hypertension and the European Society of Cardiology: ESH/ESC Task Force for the Management of Arterial Hypertension. J Hypertens.

[6] Whelton PK, Carey RM, Aronow WS, Casey DE, Collins KJ, Dennison Himmelfarb C (2018). 2017 ACC/AHA/AAPA/ABC/ACPM/AGS/APhA/ASH/ASPC/NMA/PCNA Guideline for the Prevention, Detection, Evaluation, and Management of High Blood Pressure in Adults: A Report of the American College of Cardiology/American Heart Association Task Force on Clinical Practice Guidelines. Hypertension..

[7] Lewington S, Clarke R, Qizilbash N, Peto R, Collins R (2002). Prospective Studies Collaboration. Age-specific relevance of usual blood pressure to vascular mortality: a meta-analysis of individual data for one million adults in 61 prospective studies. Lancet..

[8] Klag MJ, Whelton PK, Randall BL, Neaton JD, Brancati FL, Ford CE (1996). Blood pressure and end-stage renal disease in men. N Engl J Med.

[9] Jacobs DR, Woo JG, Sinaiko AR, Daniels SR, Ikonen J, Juonala M (2022). Childhood Cardiovascular Risk Factors and Adult Cardiovascular Events. N Engl J Med.

[10] Theodore RF, Broadbent J, Nagin D, Ambler A, Hogan S, Ramrakha S (2015). Childhood to Early-Midlife Systolic Blood Pressure Trajectories: Early-Life Predictors, Effect Modifiers, and Adult Cardiovascular Outcomes. Hypertension.

[11] Urbina EM, Khoury PR, Bazzano L, Burns TL, Daniels S, Dwyer T (2019). Relation of Blood Pressure in Childhood to Self-Reported Hypertension in Adulthood. Hypertension.

[12] Chung J, Robinson CH, Yu A, Bamhraz AA, Ewusie JE, Sanger S (2023). Risk of Target Organ Damage in Children With Primary Ambulatory Hypertension: A Systematic Review and Meta-Analysis. Hypertension.

[13] Lurbe E, Agabiti-Rosei E, Cruickshank JK, Dominiczak A, Erdine S, Hirth A (2016). 2016 European Society of Hypertension guidelines for the management of high blood pressure in children and adolescents. J Hypertens.

[14] Liu K, Colangelo LA, Daviglus ML, Goff DC, Pletcher M, Schreiner PJ (2015). Can Antihypertensive Treatment Restore the Risk of Cardiovascular Disease to Ideal Levels?: The Coronary Artery Risk Development in Young Adults (CARDIA) Study and the Multi-Ethnic Study of Atherosclerosis (MESA). J Am Heart Assoc.

[15] Health Survey for England. Health Survey for England, 2021: Data Tables, Part-2 https://digital.nhs.uk/data-and-information/publications/statistical/health-survey-for-england/2021-part-2/health-survey-for-england-2021-data-tables2023.

[16] Rana J, Oldroyd J, Islam MM, Tarazona-Meza CE, Islam RM (2020). Prevalence of hypertension and controlled hypertension among United States adults: Evidence from NHANES 2017-18 survey. Int J Cardiol Hypertens.

[17] Song P, Zhang Y, Yu J, Zha M, Zhu Y, Rahimi K (2019). Global Prevalence of Hypertension in Children: A Systematic Review and Meta-analysis. JAMA Pediatr.

[18] Dannenberg AL, Garrison RJ, Kannel WB (1988). Incidence of hypertension in the Framingham Study. Am J Public Health.

[19] Dyer AR, Liu K, Walsh M, Kiefe C, Jacobs DR, Bild DE (1999). Ten-year incidence of elevated blood pressure and its predictors: the CARDIA study. Coronary Artery Risk Development in (Young) Adults. J Hum Hypertens.

[20] Health Survey for England. Health Survey England Additional Analyses, Ethnicity and Health, 2011-2019. https://digital.nhs.uk/data-and-information/publications/statistical/health-survey-england-additional-analyses/ethnicity-and-health-2011-2019-experimental-statistics2022.

[21] World Health Organisation. Obesity and Overweight: https://www.who.int/news-room/fact-sheets/detail/obesity-and-overweight; 2021.

[22] Rimoldi SF, Scherrer U, Messerli FH (2014). Secondary arterial hypertension: when, who, and how to screen?. Eur Heart J.

[23] Grebla RC, Rodriguez CJ, Borrell LN, Pickering TG (2010). Prevalence and determinants of isolated systolic hypertension among young adults: the 1999-2004 US National Health And Nutrition Examination Survey. J Hypertens.

[24] McEniery, Yasmin CM, Wallace S, Maki-Petaja K, McDonnell B, Sharman JE (2005). Increased stroke volume and aortic stiffness contribute to isolated systolic hypertension in young adults. Hypertension.

[25] Palatini P, Rosei EA, Avolio A, Bilo G, Casiglia E, Ghiadoni L (2018). Isolated systolic hypertension in the young: a position paper endorsed by the European Society of Hypertension. J Hypertens.

[26] Li Y, Haseler E, McNally R, Sinha MD, Chowienczyk PJ (2023). A meta-analysis of the haemodynamics of primary hypertension in children and adults. J Hypertens.

[27] Nardin, Maki-Petaja C, Miles KM, Yasmin KL, McDonnell BJ, Cockcroft JR (2018). Cardiovascular Phenotype of Elevated Blood Pressure Differs Markedly Between Young Males and Females: The Enigma Study. Hypertension.

[28] Oliver-Williams C, Stevens D, Payne RA, Wilkinson IB, Smith GCS, Wood A (2022). Association between hypertensive disorders of pregnancy and later risk of cardiovascular outcomes. BMC Med.

[29] Chung F, Abdullah HR, Liao P (2016). STOP-Bang Questionnaire: A Practical Approach to Screen for Obstructive Sleep Apnea. Chest.

[30] Chung Y, de Greeff A, Shennan A (2009). Validation and compliance of a home monitoring device in pregnancy: microlife WatchBP home. Hypertens Preg.

[31] Zhang HJ, Zhang J, Wang SL, Zhang J, Teng LN, Zhang SJ (2021). Validation of the YuWell YE900 oscillometric blood pressure monitor for professional office use in adults and children according to the AAMI/ESH/ISO Universal Standard (ISO 81060-2:2018). Blood Press Monit.

[32] Stergiou GS, Palatini P, Parati G, O’Brien E, Januszewicz A, Lurbe E (2021). 2021 European Society of Hypertension practice guidelines for office and out-of-office blood pressure measurement. J Hypertens.

[33] Kulkarni S, Glover M, Kapil V, Abrams SML, Partridge S, McCormack T (2023). Management of hypertensive crisis: British and Irish Hypertension Society Position document. J Hum Hypertens.

[34] National Institute for Health and Care Excellence. Surveillance Report: 2023 surveiillance of hypertension in adults: diagnosis and management (NICE guideline NG136) and transient loss of consciousness (‘blackouts’) in over 16s (NICE guidelineCG109). Updated Feb 2023. https://www.nice.org.uk/guidance/ng136/evidence2023.

[35] Firima E, Retselisitsoe L, Leisa I, Manthabiseng M, Sematle MP, Bane M (2023). Head-to-head comparison of the WHO STEPwise approach with immediate unattended and delayed unattended automated blood pressure measurements during household-based screening: a diagnostic accuracy study in Lesotho. EClinicalMed.

[36] Wright JT, Williamson JD, Whelton PK, Snyder JK, Sink KM, Rocco MV (2015). A Randomized Trial of Intensive versus Standard Blood-Pressure Control. N Engl J Med.

[37] Myers MG, Godwin M, Dawes M, Kiss A, Tobe SW, Kaczorowski J (2010). Measurement of blood pressure in the office: recognizing the problem and proposing the solution. Hypertension.

[38] Myers MG, Kaczorowski J, Dolovich L, Tu K, Paterson JM (2016). Cardiovascular Risk in Hypertension in Relation to Achieved Blood Pressure Using Automated Office Blood Pressure Measurement. Hypertension.

[39] Salvetti M, Paini A, Aggiusti C, Bertacchini F, Stassaldi D, Capellini S (2019). Unattended Versus Attended Blood Pressure Measurement. Hypertension.

[40] Cohen JB, Denker MG, Cohen DL, Townsend RR (2019). Cardiovascular Events and Mortality in White Coat Hypertension. Ann Intern Med.

[41] Faria J, Mesquita Bastos J, Bertoquini S, Silva J, Polonia J (2020). Long-Term Risk of Progression to Sustained Hypertension in White-Coat Hypertension with Normal Night-Time Blood Pressure Values. Int J Hypertens.

[42] Sheppard JP, Fletcher B, Gill P, Martin U, Roberts N, McManus RJ (2016). Predictors of the Home-Clinic Blood Pressure Difference: A Systematic Review and Meta-Analysis. Am J Hypertens.

[43] Sheppard JP, Stevens R, Gill P, Martin U, Godwin M, Hanley J (2016). Predicting Out-of-Office Blood Pressure in the Clinic (PROOF-BP): Derivation and Validation of a Tool to Improve the Accuracy of Blood Pressure Measurement in Clinical Practice. Hypertension.

[44] Pickering TG, Eguchi K, Kario K (2007). Masked hypertension: a review. Hypertens Res.

[45] Trudel X, Brisson C, Gilbert-Ouimet M, Duchaine CS, Dalens V, Talbot D (2019). Masked hypertension incidence and risk factors in a prospective cohort study. Eur J Prev Cardiol.

[46] Chung J, Robinson C, Sheffield L, Paramanathan P, Yu A, Ewusie J (2023). Prevalence of Pediatric Masked Hypertension and Risk of Subclinical Cardiovascular Outcomes: A Systematic Review and Meta-Analysis. Hypertension.

[47] Unger N, Hinrichs J, Deutschbein T, Schmidt H, Walz MK, Mann K (2012). Plasma and urinary metanephrines determined by an enzyme immunoassay, but not serum chromogranin A for the diagnosis of pheochromocytoma in patients with adrenal mass. Exp Clin Endocrinol Diabetes.

[48] John KA, Cogswell ME, Campbell NR, Nowson CA, Legetic B, Hennis AJ (2016). Accuracy and Usefulness of Select Methods for Assessing Complete Collection of 24-Hour Urine: A Systematic Review. J Clin Hypertens (Greenwich).

[49] Faconti L, Morselli F, Sinha M, Chrysochou C, Chowienczyk PJ, British. (2021). Fibromuscular dysplasia and hypertension-a statement on behalf of the British and Irish Hypertension Society. J Hum Hypertens.

[50] Gornik HL, Persu A, Adlam D, Aparicio LS, Azizi M, Boulanger M (2019). First International Consensus on the diagnosis and management of fibromuscular dysplasia. Vasc Med.

[51] Roger, Go VL, Lloyd-Jones DM AS, Adams RJ, Berry JD, Brown TM (2011). Heart disease and stroke statistics-2011 update: a report from the American Heart Association. Circulation.

[52] Raza S, Aggarwal S, Jenkins P, Kharabish A, Anwer S, Cullington D, et al. Coarctation of the Aorta: Diagnosis and Management. Diagnostics (Basel). 2023;13:2189 10.3390/diagnostics13132189 PMC10340190 37443581

[53] Padmanabhan S, Dominiczak AF (2021). Genomics of hypertension: the road to precision medicine. Nat Rev Cardiol.

[54] Ettehad D, Emdin CA, Kiran A, Anderson SG, Callender T, Emberson J (2016). Blood pressure lowering for prevention of cardiovascular disease and death: a systematic review and meta-analysis. Lancet.

[55] Couch SC, Saelens BE, Khoury PR, Dart KB, Hinn K, Mitsnefes MM (2021). Dietary Approaches to Stop Hypertension Dietary Intervention Improves Blood Pressure and Vascular Health in Youth With Elevated Blood Pressure. Hypertension.

[56] Farpour-Lambert NJ, Aggoun Y, Marchand LM, Martin XE, Herrmann FR, Beghetti M (2009). Physical activity reduces systemic blood pressure and improves early markers of atherosclerosis in pre-pubertal obese children. J Am Coll Cardiol.

[57] Woo KS, Chook P, Yu CW, Sung RY, Qiao M, Leung SS (2004). Effects of diet and exercise on obesity-related vascular dysfunction in children. Circulation.

[58] Sundstrom J, Neovius M, Tynelius P, Rasmussen F (2011). Association of blood pressure in late adolescence with subsequent mortality: cohort study of Swedish male conscripts. BMJ.

[59] Zanchetti A, Thomopoulos C, Parati G (2015). Randomized controlled trials of blood pressure lowering in hypertension: a critical reappraisal. Circ Res.

[60] Yano Y, Stamler J, Garside DB, Daviglus ML, Franklin SS, Carnethon MR (2015). Isolated systolic hypertension in young and middle-aged adults and 31-year risk for cardiovascular mortality: the Chicago Heart Association Detection Project in Industry study. J Am Coll Cardiol.

[61] Julius S, Nesbitt SD, Egan BM, Weber MA, Michelson EL, Kaciroti N (2006). Feasibility of treating prehypertension with an angiotensin-receptor blocker. N Engl J Med.

[62] National Institute for Health and Care Excellence. Hypertension in pregnancy: diagnosis and management. NICE guideline [NG133] 2019. https://www.nice.org.uk/guidance/ng1332019. 33141539

[63] Bild DE, Bluemke DA, Burke GL, Detrano R, Diez Roux AV, Folsom AR (2002). Multi-Ethnic Study of Atherosclerosis: objectives and design. Am J Epidemiol.

[64] Howard VJ, Cushman M, Pulley L, Gomez CR, Go RC, Prineas RJ (2005). The reasons for geographic and racial differences in stroke study: objectives and design. Neuroepidemiology.

[65] Loria CM, Liu K, Lewis CE, Hulley SB, Sidney S, Schreiner PJ (2007). Early adult risk factor levels and subsequent coronary artery calcification: the CARDIA Study. J Am Coll Cardiol.

[66] Vasan RS, Massaro JM, Wilson PW, Seshadri S, Wolf PA, Levy D (2002). Antecedent blood pressure and risk of cardiovascular disease: the Framingham Heart Study. Circulation.

[67] Xie X, Atkins E, Lv J, Bennett A, Neal B, Ninomiya T (2016). Effects of intensive blood pressure lowering on cardiovascular and renal outcomes: updated systematic review and meta-analysis. Lancet.

[68] Lawson AJ, Hameed MA, Brown R, Cappuccio FP, George S, Hinton T (2020). Nonadherence to antihypertensive medications is related to pill burden in apparent treatment-resistant hypertensive individuals. J Hypertens.

[69] Constanti M, Floyd CN, Glover M, Boffa R, Wierzbicki AS, McManus RJ (2021). Cost-Effectiveness of Initiating Pharmacological Treatment in Stage One Hypertension Based on 10-Year Cardiovascular Disease Risk: A Markov Modeling Study. Hypertension.

[70] Rabbitt L, Curneen J, Hobbins A, Browne D, Joyce M, Lappin D (2024). A cost-analysis of managing secondary and apparent treatment-resistant hypertension in a specialist multidisciplinary hypertension clinic. J Hypertens.

[71] Wierzejska E, Giernas B, Lipiak A, Karasiewicz M, Cofta M, Staszewski R (2020). A global perspective on the costs of hypertension: a systematic review. Arch Med Sci.

[72] Woode ME, Wong K, Reid CM, Stowasser M, Russell G, Gwini S (2023). Cost-effectiveness of screening for primary aldosteronism in hypertensive patients in Australia: a Markov modelling analysis. J Hypertens.

[73] National Institute for Health and Care Excellence. Transition from children’s to adults’ services for young people using health or social care services. NICE guideline [NG43]Published: 24 February 2016. https://www.nice.org.uk/guidance/ng432016.

